# Gender and Weight Influence Quality of Life in Irritable Bowel Syndrome

**DOI:** 10.3390/jcm6110103

**Published:** 2017-11-01

**Authors:** LeeAnne B. Sherwin, Onyinyechi M. Ozoji, Christina M. Boulineaux, Paule V. Joseph, Nicolaas H. Fourie, Sarah K. Abey, Xuemin Zhang, Wendy A. Henderson

**Affiliations:** 1 Sinclair School of Nursing, University of Missouri-Columbia, Columbia, MO 65211, USA; sherwinl@missouri.edu; 2 Digestive Disorders Unit, Biobehavioral Branch, Division of Intramural Research, National Institute of Nursing Research, National Institutes of Health, Department of Health and Human Services, Bethesda, MD 20892, USA; onyinyechi.ozoji@nih.gov (O.M.O.); christina.boulineaux@nih.gov (C.M.B.); paule.joseph@nih.gov (P.V.J.); nicolaas.fourie@nih.gov (N.H.F.); xuemin.zhang@nih.gov (X.Z.); 3 School of Nursing & Health Studies, Georgetown University, Washington, DC 20007, USA; skabey1@gmail.com

**Keywords:** irritable bowel syndrome, quality of life, weight, gender, symptoms

## Abstract

Irritable bowel syndrome (IBS) is a common gastrointestinal disorder characterized by abdominal pain and bowel dysfunction in the absence of structural abnormality. Diagnosis can be challenging and often leads to extensive medical tests, non-effective therapeutic modalities, and reduced quality of life (QOL). Identifying factors associated with dysfunction have the potential to enhance outcomes. Participants with IBS (*n* = 41) and healthy volunteers (*n* = 74) were recruited into this cross-sectional, descriptive, natural history protocol at the National Institute of Health, Clinical Center. Demographic characteristics were self-reported. QOL was assessed with the Irritable Bowel Syndrome Quality of Life (IBS-QOL) questionnaire. Statistical analysis included descriptive statistics, factorial ANOVA, and multiple regression. Individuals with IBS reported lower QOL scores across all QOL-subscales compared to healthy controls. Normal-weight women and overweight men with IBS reported the greatest QOL impairment. Body fat percent had confounding effects on the relationship between IBS and QOL. The disparity between QOL scores in participants with IBS by both gender and weight groups may reflect different social pressures perceived by normal and overweight women and men. These findings enhance the recognition of the disparities in patients with chronic symptoms and thereby lead to personalized assessment and interventions to improve their QOL.

## 1. Introduction

Irritable bowel syndrome (IBS) is a common functional gastrointestinal disorder estimated to affect 20% of the United States population and 11% of individuals globally [1]. Individuals with IBS experience abdominal pain and disruption of their bowel habits. Currently, the diagnosis of IBS is based on up-to-date guidelines using Rome criteria which determines IBS subtypes based on stool consistency patterns, such as diarrhea predominant (IBS-D), constipation predominant (IBS-C), or mixed symptoms of both constipation and diarrhea (IBS-M) [2]. The pathophysiology of IBS is not entirely understood. Currently, the syndrome’s etiology is thought to be multifactorial in origin. Factors such as alterations in gut motility, pain perception, intestinal permeability, and the gut microbiome, in addition to notable influences from psychosocial factors, diet, genetics, and inflammation, have all been identified as contributors to the development of IBS [3,4,5].

Women have the highest incidence of IBS, with rates that are 1.5- to 3-fold higher than those of men [6]. The underlying mechanism for this sex disparity is not well understood. Variation in prevalence can be attributed to both sex- and gender-related differences. Sex refers to the biological characteristics that identify men and women, whereas gender refers to roles assigned by society that are believed to be belonging to men and women [7]. These sex- and gender-related differences translate into variation between men and women in both healthcare-seeking behavior and the sociocultural characteristics of individuals who suffer with IBS. Much of the IBS research to date has not rigorously tested for gender differences in regard to perceived quality of life (QOL), despite existing evidence of significant impairment in QOL among IBS sufferers [8].

Health consequences resulting from added weight range from premature death to chronic conditions that result in reduced QOL. Obesity is one of today’s major public health concerns. The World Health Organization considers obesity to be an escalating global health problem [9]. In the past several years, studies have shown associations between obesity and upper gastrointestinal symptoms, such as reflux [10,11,12,13]; however, few have examined the relationship of obesity and lower gastrointestinal symptoms, such as chronic abdominal pain, as experienced by those with IBS [14].

This study thus aimed to evaluate the relationship between gender, Body Mass Index (BMI), body fat percent, and QOL as experienced by carefully phenotyped individuals with IBS. We hypothesized that overweight classification would be associated with impaired quality of life as experienced by individuals with IBS.

## 2. Experimental Section

The sample consisted of 125 male and female participants, ages 13 to 45 years. Ten participants did not complete the study, resulting in the use of 115 participants in the analysis. At the time of this study, all participants were assessed with either adult or pediatric Rome III diagnostic criteria for the establishment of an IBS diagnosis, fasted for 8 h, and had a scheduled morning clinical visit. Female participants completed the protocol between days 3 and 10 of their menstrual cycle to control for hormonal variation. Although participants were self-referred, each was carefully phenotyped through history and physical examination that included screening blood work and stool to exclude gastrointestinal pathologies. Exclusion criteria included the presence of any self-reported or verified organic disease (gastrointestinal, pulmonary, neurologic, renal, endocrine, or gynecological pathology) and the use of daily medication for any chronic medical condition treatment.

Cross-sectional analysis was completed on data from participants recruited under a natural history protocol (Clinicaltrial.gov #NCT00824941) conducted at the Hatfield Clinical Research Center at the National Institutes of Health, Bethesda, Maryland. Body mass index, body fat percentage, and questionnaires were collected during outpatient visits that took place from January 2009 to December 2015. The study was compliant with good clinical practice guidelines and the Declaration of Helsinki.

### 2.1. Sociodemographic Questionnaire

Data on gender, race, marital status, years of education, and work status were obtained via self-report using the Sociodemographic Questionnaire developed by the Center for Research in Chronic Disorders, University of Pittsburgh School of Nursing (1999).

### 2.2. Body Mass Index (BMI)

BMI was calculated by first measuring the patients’ weight in triplicate and calculating the average. Next, the height was measured in duplicate and then averaged. Height and weight averages were used to determine BMI, calculated as weight in kilograms divided by height in meters squared. Weight status was classified as follows: normal weight (BMI 18.5–24.9 or 5th to <85th percentile for pediatric participants) and overweight (≥BMI 25.0 or ≥85th percentile for pediatric participants).

### 2.3. Body Fat Percentage

A trained registered nurse completed whole-body air displacement plethysmography technology (Bod Pod^TM^) on all patients to determine body fat percentage and confirm BMI. The Bod Pod^TM^ is a body composition assessment system that divides participants’ mass measurements by their volume measurements to determine an average body density. The Bod Pod^TM^ is powerful because of its capacity to measure volume with high specificity, as it takes multiple volume measurements of the subject’s body as well as their lung volume, which is subtracted from the overall volume. The Bod Pod^TM^ then uses a built-in algorithm to calculate percent body fat and percent lean mass of the individual.

### 2.4. Quality of Life Measure (IBS-QOL)

The QOL of the participants was assessed using the Irritable Bowel Syndrome-Quality of Life (IBS-QOL) questionnaire. Eight QOL subscales were reported: Body Image, Dysphoria, Food Avoidance, Health Worry, Interference with Activity, Relationship, Social Reaction, and Sexual Activity. The IBS-QOL is a self-report measure that is used to assess the impact of IBS on QOL [15]. The IBS-QOL score was determined according to the method established by Patrick et al. [16]: individual responses to the 34 items were summed and averaged for a total score and then transformed to a 0–100 scale to simplify interpretation, with lower scores indicating greater QOL impairment.

Clinical and demographic data were analyzed with Statistica version 12.0 (StatSoft, Tulsa, OK, USA) and RStudio V1.0.136 (RStudio, Boston, MA, USA), with significant *p*-values set at alpha ≤ 0.05. Group differences (gender, IBS, and weight group) were also assessed using Students *t-*Test or Mann Whitney *U* test. Data are presented as mean ± standard deviation for continuous variables and counts and percentages for categorical variables. Data was also assessed by Kolmogorov–Smirnov tests for normality. The study was designed to detect the minimum mean difference in quality of life scores between individuals with IBS and healthy controls. The sample size required for detecting this significant difference was 98, at a significance level of 0.05 and a power of 80%. We took into consideration a 20% attrition rate and enrolled 125 subjects. Ten participants did not complete the study, which resulted in 115 participants in the analysis that exceeded the minimum sample size.

In a linear model developed for the outcome measure of different QOL subscales, predictors included gender, IBS status, and body fat percentage, as well as separate interaction terms between each variable. Variables were removed from the model when there was neither a main effect nor significant interactions with other variables. IBS status, body fat percentage, and an IBS*body fat percentage interaction term were included as predictors in a multiple regression model for the prediction of each QOL subscale outcome. When the interaction term did not significantly predict QOL, it was removed from the model.

## 3. Results

### 3.1. Subjects

One hundred and fifteen male and female participants between the ages of 13–45 years participated in the study. Healthy controls (*n* = 74) were defined as individuals who did not report any gastrointestinal (GI) symptoms and had no known organic disease. IBS participants (*n* = 41) consisted of individuals who met either adult or pediatric Rome III IBS diagnostic criteria [17]. The majority of participants resided in the greater Washington, DC area, including the surrounding suburbs in Maryland and Virginia. Descriptive data of the participants is displayed in Table 1. There were no significant differences regarding age, gender, and weight between participants with IBS and healthy controls.

### 3.2. Body Mass Index

Body mass index did not differ between IBS and healthy control groups. Overall BMI ranged from 18.6–46.8 with a mean of 26.90 (SD = 6.41) for individuals with IBS and 26.40 (SD = 5.84) for healthy controls.

### 3.3. Quality of Life

Low QOL scores were reported by 84% of men and 92% of women with IBS. Table 2 shows the scores of the QOL and as well as QOL subscales. Individuals with IBS reported significantly lower total QOL scores (M = 76.91 ± 22.11) than healthy controls (HC) (M = 98.90 ± 5.13, *p* < 0.05). When groups were compared based on body weight, overweight males with IBS reported lower total QOL scores (M = 77.21 ± 17.33) compared to overweight males without IBS (M = 99.60 ± 1.20). Across all groups, normal weight females with IBS reported the lowest total QOL score (M = 74.60 ± 18.63). Examination of QOL subscales revealed that males with IBS had the greatest impairment in the food avoidance subscale (M = 66.67 ± 27.86). Females with IBS had significant impairment of all of QOL subscale measures; however, the health worry (M = 64.13 ± 27.46) and food avoidance (M = 66.67 ± 28.98) subscales were found to have the greatest impairment.

### 3.4. Prediction of QOL Subscales by IBS Status and Body Fat Percentage

Linear regression analyses were used to evaluate the effect of IBS status and body fat percentage on QOL (Table 3). IBS status and body fat percentage also interacted to affect different QOL subscales in the study’s participants, justifying the inclusion of the IBS*body fat interaction term in the prediction model. Having IBS significantly predicted lower QOL subscale scores for dysphoria (*p* ≤ 0.001), food avoidance (*p* ≤ 0.001), social reaction (*p* ≤ 0.001), and relationships (*p* ≤ 0.001). While higher body fat percent alone did not predict a lower QOL in any of the dimensions listed, having a higher body fat percent while also having IBS predicted lower QOL overall (*p* ≤ 0.01). This interaction also predicted lower QOL scores in interference with activity (*p* ≤ 0.05), body image (*p* ≤ 0.001), health worry (*p* ≤ 0.001), and sexual satisfaction (*p* ≤ 0.01).

## 4. Discussion

The aim of this study was to evaluate the relationship between gender, BMI, and QOL as experienced in carefully phenotyped individuals with IBS compared to healthy controls. Consistent with our hypothesis, individuals with IBS reported greater impairment of their QOL as compared to healthy controls. Both males and females with IBS experienced low QOL scores; however, females reported the lowest. Additionally, a higher body fat percent was found to predict lower QOL overall in those with IBS.

The results of this study demonstrate significant impairment of the QOL among individuals who suffer from IBS. We found in this cohort that both males and females with IBS reported similar impairment of all eight subscales as well as overall QOL. For instance, scores on the food avoidance subscale demonstrated significant QOL impairment for IBS sufferers of both genders, reflecting similar results found in previous studies [18,19]. In our previous work in a separate study population, we found that individuals with IBS who catastrophized often reported significantly greater impairment of the food avoidance subscale compared to those who did not catastrophize [8]. Additionally, in a study of adults both with and without IBS that underwent a dietary guidance intervention, Østgaard and colleagues [20] found that at a two-year follow-up, individuals with IBS reported improved QOL scores as well as a decrease in symptoms due to the avoidance of aggravating foods. In the same study, IBS participants who did not receive the dietary guidance intervention did not demonstrate score improvements. Furthermore, individuals with IBS demonstrated higher selectivity in their food choices; these individuals consumed less alcohol and fewer fermentable oligosaccharides, disaccharides, monosaccharides, and polyols (FODMAPs) foods. Although overall QOL was better in the individuals with IBS subject to dietary education intervention, their food avoidance-specific subscale scores were not significantly different than those with IBS who did not undergo the intervention (59.4 ± 2.6 vs. 59.4 ± 3.3, *p ≥* 0.05). These findings reflect our results that IBS has a significant impact on patients’ eating habits resulting in persistent QOL impairment.

Compared to males, females reported lower QOL scores in seven of the eight subscales. For females in our study, the lowest subscale score was found in the health worry category. The scores reported were similar to those reported by Paré and colleagues [21] in a natural study in Canada of 1555 patients with IBS, 85% of which were females; participants from that study reported significant impairment of their QOL. Similar to our study, participants reported significant impairment of the food avoidance (M = 51.8 ± 29.6) and health worry (M = 59.3 ± 26.2) subscales. Findings from both our study and the study by Paré and colleagues [21] suggest that food avoidance and health worry, in addition to the remaining QOL categories, are consistently lower for female patients seeking care in the community.

Use of the IBS-specific QOL measure captures concerns that are specifically pertinent to IBS, and the disease-specific QOL scores found in our cohort reflect the high burden of IBS in these individuals. The reduction in QOL of both males and females with IBS in our study was significant; however, weight inconsistently influenced QOL in these participants. In those with IBS, overweight males and normal weight females reported considerable QOL impairment, with the greatest impairment experienced by normal weight females. Although researchers have pursued an explanation for the association of weight and IBS, the link remains unclear [22,23]. A considerable numbers of studies have examined potential mechanisms by which weight contributes to IBS symptoms and to the development of the syndrome [14,24,25,26]. In contrast to Talley and colleagues [13] and Delgado-Aros and colleagues [22], our study finds that individuals’ weight categorization report inconsistent impacts of their IBS on their QOL. It is unclear why normal weight women and overweight men report the greatest impairment of their QOL. We suspect that psychosocial, cultural, and healthcare-seeking behaviors are potential contributors to this result; future research should examine the role these factors play in shaping QOL in these subpopulations.

A post hoc analysis of body fat percentage was completed to help explain the effect of weight in IBS. We found an interactive effect of IBS and body fat percentage on QOL. Specific subscales that included both physical and emotional functioning were impaired. In previous work [27], we compared study participants categorized according to both body composition (i.e., body fat percentage) and BMI, and observed gene expression differences between the weight classification methods. Current evidence demonstrates that both BMI and body fat percentage are important indicators of QOL. These insights present a worrisome outlook for individuals with IBS who are also overweight or who have increased body fat.

Several aspects of this study add strength to existing analyses on the subject; most notably, the obesity index used to evaluate the weight status in participants was not measured solely by calculated BMI. Instead, BMI measures were confirmed with objective whole-body air displacement plethysmography (Bod Pod^TM^). In addition, trained study investigators used adult or pediatric Rome III criteria to confirm the IBS diagnosis. Although there were several strengths to our study, it is important to mention the potential limitations. Participants were self-referred, which may have resulted in selection bias. In addition, this recruitment method resulted in high geographic homogeneity among participants; the area where most participants took residence may not reflect the more general IBS population. Despite these limitations, this study provides useful information that may contribute to current understandings of gender and weight differences in individuals with IBS.

## 5. Conclusions

IBS has clear implications on the QOL of individuals experiencing this condition. Results from this study indicate that both male and female participants with IBS report significant impairment in their QOL. Furthermore, body fat percentage was found to have an incremental effect on both total QOL and specific QOL subscales. Potential targeted interventions in individuals that suffer from significant IBS symptoms include increasing lean body mass by decreasing body fat percentage, thereby improving body image, decreasing health worry, experiencing less interference with activity, and improving of self-perceptions of sexual activity and desire.

## Figures and Tables

**Table 1:** Demographic characteristics of participants.

Variable	Overall (*n* = 115) *n* (%)	IBS (*n* = 41) *n* (%)	Healthy (*n* = 74) *n* (%)
Age (M ± SD)	28.2 ± 7.9	28.0 ±8.1	28.3 ± 7.8
Sex			
Male	57 (50)	18 (44)	39 (55)
Female	58 (50)	23 (56)	35 (47)
Race			
African American	35 (30)	12 (29)	23 (31)
Asian	16 (14)	5 (12)	11 (15)
Caucasian	54 (47)	21 (51)	33 (46)
Other	10 (8)	3 (7)	7 (10)
Weight group			
Normal weight	57 (50)	19 (46)	38 (54)
Overweight	58 (50)	22 (54)	36 (51)
Percent Body Fat			
Male	23.07% (SD ± 9.66)	23.32% (SD ± 11.33)	22.95% (SD ± 8.99)
Female	34.02% (SD ± 9.00)	34.30% (SD ± 8.30)	33.84% (SD ± 9.51)

IBS = Irritable Bowel Syndrome.

**Table 2:** Quality of life scores of participants with IBS and healthy controls (total and subscales).

Variable	IBS Male Mean (SD) *n* = 18	IBS Female Mean (SD) *n* = 23	Healthy Male Mean (SD) *n* = 39	Healthy Female Mean (SD) *n* = 35	IBS Normal Weight Mean (SD) *n* = 19	IBS Overweight Mean (SD) *n* = 22	Healthy Normal Weight Mean (SD) *n* = 38	Healthy Overweight Mean (SD) *n* = 36
IBS-QOL total score	78.80 (±16.87)	76.53 (±17.12)	98.87 (±3.62)	98.71 (±2.47)	77.21 (±17.91)	77.81 (±16.27)	98.35 (±4.09)	99.26 (±1.44)
Subscales								
Body image	84.38 (±20.14)	75.54 (±18.74)	99.67 (±1.40)	99.10 (±2.69)	82.33 (±15.68)	78.41 (±20.57)	99.51 (±1.71)	99.31 (±2.49)
Dysphoria	77.43 (±19.01)	76.49 (±24.68)	98.71 (±6.56)	99.10 (±2.89)	76.48 (±23.80)	77.28 (±21.09)	98.19 (±7.03)	99.65 (±1.24)
Food avoidance	66.67 (±27.86)	66.67 (±28.98)	99.15 (±3.20)	97.86 (±6.18)	61.84 (±28.50)	70.83 (±27.79)	97.81 (±6.33)	99.31 (±2.33)
Health worry	78.24 (±20.64)	64.13 (±27.46)	99.57 (±1.86)	98.33 (±6.33)	69.74 (±29.03)	70.83 (±22.53)	98.25 (±6.17)	99.77 (±1.39)
Interference with activity	77.58 (±20.12)	81.21 (±14.73)	98.90 (±4.71)	98.78 (±2.99)	82.33 (±15.68)	77.27 (±18.39)	98.68 (±4.94)	99.01 (±2.65)
Relationships	86.58 (±13.75)	86.23 (±15.20)	98.50 (±4.63)	98.10 (±4.56)	85.53 (±16.63)	87.12 (±12.53)	98.25 (±3.95)	98.38 (±5.20)
Sexual activity	88.19 (±25.89)	78.26 (±24.48)	99.04 (±6.00)	98.57 (±6.62)	83.55 (±23.22)	81.82 (±27.47)	98.25 (±3.95)	98.38 (±5.20)
Social reaction	77.08 (±21.65)	77.98 (±20.63)	97.76 (±6.49)	98.92 (±2.83)	74.03 (±22.75)	80.68 (±18.99)	97.70 (±6.40)	98.96 (±3.17)

IBS = Irritable Bowel Syndrome, SD = Standard Deviation, IBS-QOL = Irritable Bowel Syndrome-Quality of Life measure.

**Table 3:** Multiple regression model for quality of life subscales.

Quality of Life Total and Subscales	Predictor	Estimate	*p*-Value
Overall (Total-Quality of Life)	IBS	−7.865	0.16
	Body fat percentage	0.002	0.98
	IBS*Body fat percentage	−0.471	0.01
Body image	IBS	−0.48	0.94
	Body fat percentage	−0.05	0.67
	IBS*Body fat percentage	−0.71	0.001
Dysphoria ^1^	IBS	−22.430	0.001
	Body fat percentage	−0.078	0.52
Food avoidance ^1^	IBS	−21.20	0.001
	Body fat percentage	−0.03	0.75
Health worry	IBS	−4.10	0.62
	Body fat percentage	−0.03	0.84
	IBS*Body fat percentage	−0.85	0.001
Interference with activity	IBS	−2.66	0.64
	Body fat percentage	0.02	0.88
	IBS*Body fat percentage	−0.58	0.05
Relationships ^1^	IBS	−12.03	0.001
	Body fat percentage	−0.14	0.09
Sexual	IBS	4.91	0.57
	Body fat percentage	−0.00	0.99
	IBS*Body fat percentage	−0.73	0.01
Social reaction ^1^	IBS	−21.20	0.001
	Body fat percentage	−0.04	0.75

^1^ IBS*body fat interaction was non-significant and therefore was removed from the model.
